# Transcriptional Regulation of the Bovine Fatty Acid Transport Protein 1 Gene by Krüppel-Like Factors 15

**DOI:** 10.3390/ani9090654

**Published:** 2019-09-05

**Authors:** Zhidong Zhao, Hongshan Tian, Bingang Shi, Yanyan Jiang, Xiu Liu, Jiang Hu

**Affiliations:** College of Animal Science and Technology & Gansu Key Laboratory of Herbivorous Animal Biotechnology, Gansu Agricultural University, Lanzhou 730070, China (Z.Z.) (H.T.) (B.S.) (Y.J.) (X.L.)

**Keywords:** bovine, *SLC27A1* gene, Krüppel-like factors 15, unsaturated fatty acid, 5′RACE

## Abstract

**Simple Summary:**

The nutritional value and qualities of beef are enhanced when the unsaturated fatty acid content is increased. Fatty acid transport protein 1 (FATP1), also called SLC27A1, an integral membrane protein that facilitates long-chain fatty acid influx, is involved in the genetic network for oleic acid synthesis in beef. Polymorphisms in bovine *SLC27A1* gene are most significantly associated with oleic acid. Its expression exhibits significant positive correlations with bovine intramuscular fat content in the longissimus thoracis muscle. However, the transcription factors that contribute to the control and regulation of its expression have not been characterized extensively. In this study, we determined the tissue distribution of *SLC27A1* mRNA and found that bovine *SLC27A1* was highly expressed in subcutaneous adipose tissue and the longissimus thoracis muscle. Furthermore, we analyzed the molecular mechanisms involved in *SLC27A1* regulation and found that the transcriptional activity of *SLC27A1* gene was dependent on KLF15 transcription factor. These results may lead to an enhanced understanding of the regulation of *SLC27A1* expression in other models, as well as provide new insights into the regulatory mechanism and biological functions of the *SLC27A1* gene in determining the lipid composition in beef.

**Abstract:**

Oleic acid is a major monounsaturated fatty acid, which accounts for about 33% of the fatty acid content in beef and is considered to have the least negative effect on serum cholesterol levels. Fatty acid transport protein 1 (FATP1), an integral membrane protein that facilitates long-chain fatty acid (LCFA) influx, is involved in the genetic network for oleic acid synthesis in beef. Its expression exhibits significant positive correlations with intramuscular fat (IMF) content in the longissimus thoracis. However, the expression mechanism of *SLC27A1* or *FATP1* is still unclear. To elucidate the molecular mechanisms involved in bovine *SLC27A1* regulation, we cloned and characterized the promoter region of *SLC27A1*. By applying 5′-rapid amplification of cDNA end analysis, we identified two alternative splice variants of this gene. Using a series of 5′ deletion promoter plasmids in luciferase reporter assays, we found that the core promoter was 96 base pairs upstream from the transcription initiation site. Electrophoretic mobility shift assay combined with a site-directed mutation experiment demonstrated that KLF15 binding to the promoter region drives the *SLC27A1* transcription. KLF15 plays an essential role in adipogenesis and skeletal muscle lipid flux. Thus, these results might provide further information on the regulatory roles of *SLC27A1* gene in mediating the lipid composition in beef.

## 1. Introduction

Raising the levels of unsaturated fatty acids in beef is an optimal means of improving its nutritional value and its overall quality for consumption. Oleic acid (C18:1n9) makes up roughly one-third of all fatty acids present in beef, and as a monounsaturated fat it has the least adverse impact on levels of serum cholesterol of all types of fatty acids found within beef [1]. Fatty acid transport protein 1 (FATP1), or *SLC27A1*, is a protein found within the membrane of cells, where it facilitates the influx of long-chain fatty acids (LCFA). It is also involved in the synthesis of oleic acid making it a viable target for efforts in improving beef quality and nutritional value [2]. Insulin has been shown to drive the translocation of *SLC27A1* from within cells to the cell surface where it can help in enhancing the intake of LCFAs [3]. The nuclear orphan receptor TR4 has been shown to promote the expression of *SLC27A1* within 3T3-L1 adipocytes, thereby, driving the accumulation of lipids [4]. In studies investigating gain-of-function experiments, *SLC27A1* was shown to mediate adipocyte fatty acid (FA) uptake [5]. When *SLC27A1* activity or expression was disrupted in a murine model system, it led to an increase in plasma FA levels that coincided with a drop in the FAs within the skeletal muscle and adipose tissue [6,7]. Adipocytes and tissues that rapidly metabolize FAs such as the skeletal and heart muscles express significant levels of *SLC27A1*, whereas it is absent in the hepatic tissue [8]. In 3T3-L1 preadipocytes, *SLC27A1* is only expressed at low levels, with a marked elevation in its expression occurring only after their conversion to an adipose phenotype [5,9]. This is consistent with observed increases in the uptake of oleic acid that occurs during the differentiation of preadipocytes [10]. When *SLC27A1* is depleted in mice, there is a reduced uptake of FAs and triglycerides and deposition of these compounds into the adipose tissue [6,11]. The gene encoding *SLC27A1* encompasses a 40 kb region, encoding 13 exons [8], and is mapped in the bovine chromosome 7) (BTA 7) [12], in which multiple quantitative trait loci known to be relevant to fat-associated traits are located [13]. Bovine *SLC27A1* mutations are markedly associated with oleic acid (C18:1n9) [2], and the extent to which this gene is expressed is positively correlated with the intramuscular fat (IMF) levels in the longissimus thoracis muscle [14].

KLF15 is a zinc-finger DNA-binding factor that belongs to the Spl-like/KLF family, which is involved in regulatory roles governing the metabolism of glucose, lipids, and amino acids [15,16,17]. As 3T3-L1 cells differentiate, they significantly upregulate KLF15, and its overexpression is sufficient to induce adipogenesis in these cells [18]. KLF15 promotes the transcription of *ACSL1*, and has the potential to regulate the IMF levels in the bovine skeletal muscle [19]. Chromatin immunoprecipitation studies have confirmed that KLF15 binds near the *SLC27A1* promoter in skeletal and cardiac muscle tissues [20].

While *SLC27A1* is known to play key roles in regulating bovine skeletal muscle’s IMF content, the transcriptional processes regulating its expression are not completely understood. In the present study, we highlighted two splicing variants of this gene in the longissimus thoracis muscle. We further observed high expression of *SLC27A1* in both the subcutaneous adipose tissue and in the longissimus thoracis muscle. We additionally found that KLF15 was an essential transcriptional regulator of *SLC27A1* expression. Further efforts to understand the transcriptional mechanisms governing *SLC27A1* may yield insights valuable to regulating the lipid composition within the beef.

## 2. Materials and Methods

All animal experiments were conducted in accordance with the guidelines for the care and use of experimental animals established by the Ministry of Science and Technology of the People’s Republic of China (Approval number 2006-398) and were approved by the Animal Care Committee of Gansu Agricultural University, Lanzhou, China.

### 2.1. Tissue Expression Profiling

Three adult Qinchuan cattle served as a source of 14 tissue samples, from which total RNA was isolated using the Total RNA kit (Tiangen, Beijing, China). cDNA was generated through use of the PrimeScript™ RT reagent Kit (TaKaRa, Dalian, China), and individual animal sample tissue cDNA were pooled before performing a qPCR analysis using a SYBR Green PCR Master Mix kit (TaKaRa) with a 7500 System SDS V 1.4.0 (Applied Biosystems, Foster City, CA, USA). Table S1 (Supplementary Material) contains the primers used in the present study. All gene expressions were normalized with of *β-actin (ACTB)* expression, and the 2^−ΔΔCt^ method was used to compare gene expressions [21].

### 2.2. Rapid Amplification of cDNA Ends (5′ RACE)

The BD SMARTTM RACE cDNA amplification kit (Clontech Inc, Mountain View, CA, USA) was used to identify the bovine *SLC27A1* transcriptional start site (TSS) based upon the manufacturer’s directions. In short, 1 μg longissimus thoracis RNA underwent PowerScript RT (Clontech Inc, Mountain View, CA, USA) reverse transcription, after which Universal Primer A Mix (UPM) (Clontech Inc, Mountain View, CA, USA) was added for PCR amplification along with appropriate primers (Table S1) that were specific for *SLC27A1* exons 2, 3, and 4. The amplicons from this PCR reaction were subjected to a 20-fold dilution, followed by 2% agarose gel separation in a gel supplemented with 0.6 μg/mL ethidium bromide for UV visualization of the DNA. After purification, the amplified sequences were cloned into T-Vector pMD19 (simple) (TaKaRa, Dalian, China) and 20 clones were subject to sequencing.

### 2.3. Construct Generation

Primers specific for the 2 kb region upstream of the bovine *SLC27A1* TSS were used in order to amplify a 2034 bp PCR product that was separated via gel extraction. This was then cloned into the T-Vector pMD19 (simple) vector followed by submission to GenBank (Submission No. KU215705). Luciferase reporters were generated by digesting the promoter-containing T-Vector pMD19 (simple) constructs using Sac1 and Xho1 (TaKaRa, Dalian, China), and finally by ligating this promoter into the pGL3-basic vector, yielding the pGL3-1856 plasmid construct. We further generated pGL3–1558, –1261, –955, –640, –387, and –96 plasmids in which unidirectional regions of this promoter were deleted through the use of specific primers containing Sac1 and Xho1 restriction sites. The QuickChange Site-Directed Mutagenesis Kit (Stratagene, La Jolla, CA, USA) was used to introduce site-specific mutations. Transcription factor binding sites in the *SLC27A1* promoter were analyzed using MatInspector tool (http://www.genomatix.de), and this same tool was used to ensure that novel binding sites were not introduced upon site-directed mutagenesis. Bidirectional sequencing was conducted for all constructs (Jinsirui, Nanjing, China).

### 2.4. Cell Culture and Transfection

The murine C2C12 and 3T3L1 cell lines were grown using DMEM (Invitrogen, Carlsbad, CA, USA) containing 10% FBS (PAA, Kremplstraße, Austria), 4500 mg/L glucose, and penicillin/streptomycin in a humidified 37 °C, 5% CO_2_ incubator.

For transfection, cells were added to 24-well plates (1.2 × 105 per well) overnight in antibiotic-free media in order to achieve 80–90% confluency. The X-tremeGENE HP DNA transfection reagent (Roche, Basel, Switzerland) was then used for transfection with appropriate plasmids based on manufacturer’s directions. The transfection reagent was then combined with opti-MEM (6 μg/150 μL) (Invitrogen) for 5 min, after which it was mixed with appropriate plasmid concentrations for 30 min (2.4 μg/150 μL for pGL3 and 0.03 μg/150 μL for pRL-TK). Finally, 100 μL of this mixture was then added per well for 48 h, after which cells were washed in PBS and lysed with passive lysis buffer (Promega, Madison, WI, USA). The Dual Reporter assay system (Promega, Madison, WI, USA) and NanoQuant Plates™ (TECAN, infinite M200PRO, Männedorf, Switzerland) were used to assess luciferase activity, with Renilla luciferase activity used for normalization.

### 2.5. Electrophoretic Mobility Shift Assays (EMSA)

NA Nuclear Extract Kit (Active Motif, Carlsbad, CA, USA) was used to isolate nuclei of C2C12 cells (ATCC, Rockefeller, Maryland, USA) according to the manufacturer’s instructions. Bradford assay (Bio-Rad, Hercules, CA, USA) was used to quantify protein levels in samples. EMSA DNA probes (Table S1) were synthesized (Invitrogen) and 5′ biotinylated. Next, 10 μg nuclear protein was mixed for 15 min with 2 μL 10× binding buffer and 1 μL poly (dI.dC) in 20 μL in ice-cold conditions, after which labeled probes (200 fmol) were added for 20 min at room temperature. In competition experiments, excess unlabeled or mutant probes were added, 15 min prior to the addition of these labeled probes. In super-shift experiments, 10 μg anti-KLF15 (Santa Cruz, CA, USA) was added in ice-cold conditions for 30 min before labeled probe addition. After this, 6% non-denaturing polyacrylamide gel electrophoresis (PAGE) was carried out for complex resolution using 0.5× TBE (Solarbio, Beijing, China) for 1 h.

## 3. Results

### 3.1. Assessment of the Expression of SLC27A1 in Bovine Tissues

**Figure 1:**
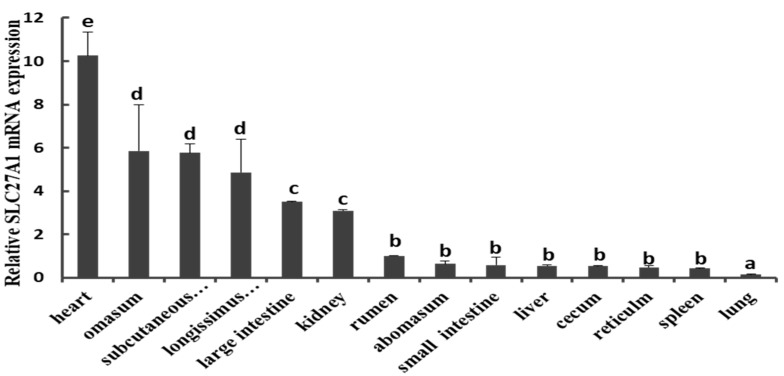
Expression patterns of *SLC27A1* in bovine tissues and organs. *SLC27A1* mRNA expression was normalized against the housekeeping gene β-actin (ACTB) expression and expressed relative to the gene expression in the rumen. Each column value represents the mean ± standard deviation based on three independent experiments; n = 3. The error bars denote the standard deviations. The different lower-case letters indicate significant difference (*P* < 0.05).

We first measured the expression of *SLC27A1* via qPCR using tissue samples collected from the heart, omasum, subcutaneous adipose tissue, longissimus thoracis muscle, large intestine, kidney, rumen, abomasum, small intestine, liver, cecum, reticulum, spleen, and lung of three cattle. This analysis revealed a marked expression of *SLC27A1* in many tissues, with a particularly high baseline expression in the adipose tissue and in the longissimus thoracis muscle, whereas levels were markedly lower in the liver, spleen, lung, small intestine, and other tissues (Figure 1).

### 3.2. SLC27A1 TSS Identification

**Figure 2:**
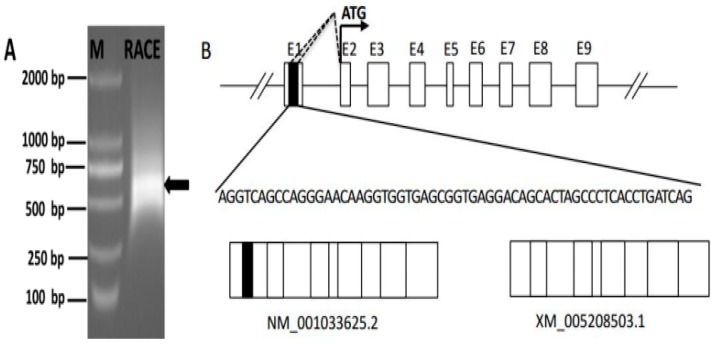
Identification of the *SLC27A1* transcriptional start site. (**A**) Nested PCR was used to generate *SLC27A1* 5′ RACE products that were subjected to agarose gel electrophoretic separation, with arrows corresponding to the amplicons. (**B**) The initiation sites for the two *SLC27A1* mRNA variants identified via 5′ RACE are shown along with a partial genomic structure for this gene, and the initiation codon ATG are indicated with arrows. White boxes identify constitutive exons, while the black box highlights the alternatively spliced region. Introns are indicated by solid lines and dashed lines indicating potential splicing variations.

We next conducted 5′-RACE to identify the *SLC27A1* TSS, by performing two rounds of PCR amplification with both an antisense and a nested primer (SLC27A1-GSP1 and SLC27A1-GSP2; Table S1). This led to the identification of a 531 bp amplicon (Figure 2A). Products from the second of these 5′-RACE interactions were cloned into the T-Vector pMD19 (simple) vectors after which 20 colonies were sequenced. A total of 4/20 clones showed 100% sequence identity to the predicted *SLC27A1* sequence (XM_005208503.1) while the rest were 100% identical to the *SLC27A1* gene sequence (NM_001033625.2) (Figure 2B). These two variant sequences both had a TSS within exon 2, suggesting that they shared a promoter and that their transcription is not under the control of an alternative promoter. Multiple initiation sites were present in a 100 bp region within this exon, with alternative splicing results suggesting that these sites are present in the region from bases 323-228 from the variant 1 initiation codon ATG, and in a region from bases 314-291 from the variant 2 ATG codon.

### 3.3. SLC27A1 Promoter Sequencing and Isolation

**Figure 3:**
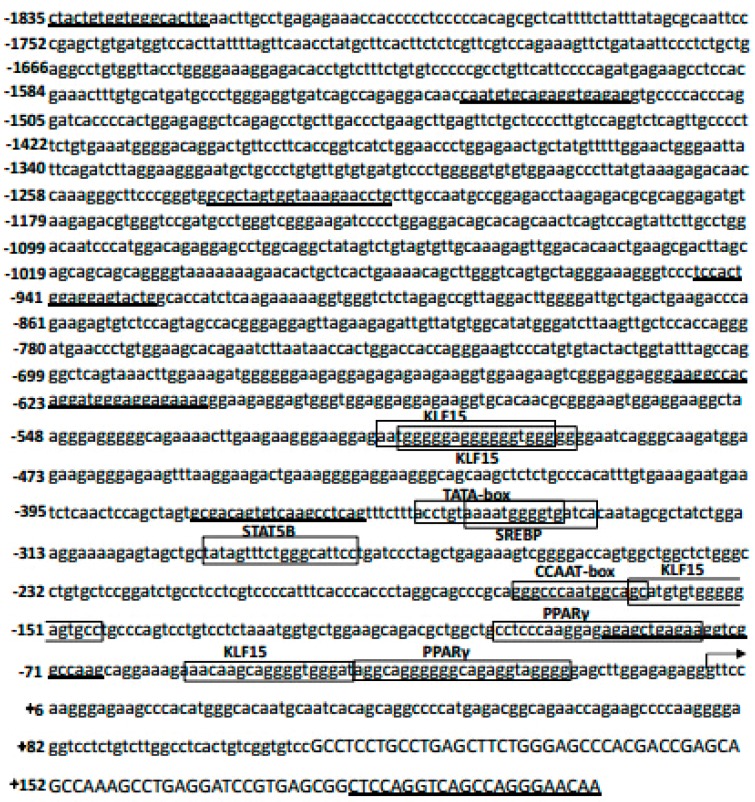
Sequence of the bovine *SLC27A1* promoter and a segment of exon 1. The *SLC27A1* TSS is marked with an arrow, with exon 1 indicated by capital letters. Unidirectional deletion primers are underlined, and boxes indicate potential sites of transcription factor binding.

In order to explore the potential promoter regulatory elements governing the transcription of *SLC27A1*, we used Matlnspector tool (http://www.genomatrix.com) with a >90% cut-off value to examine this region, revealing multiple regulatory elements with potential binding sites for KLF15. Sterol regulatory element-binding protein (SREBP), signal transducer and activator of transcription 5B (STAT5B), and peroxisome proliferator-activated receptor γ (PPARγ) binding sites were also detected (Figure 3). This 5′ region also contained TATA and CCAAT box elements, as is common for mammalian type-2 promoters. 

**Figure 4:**
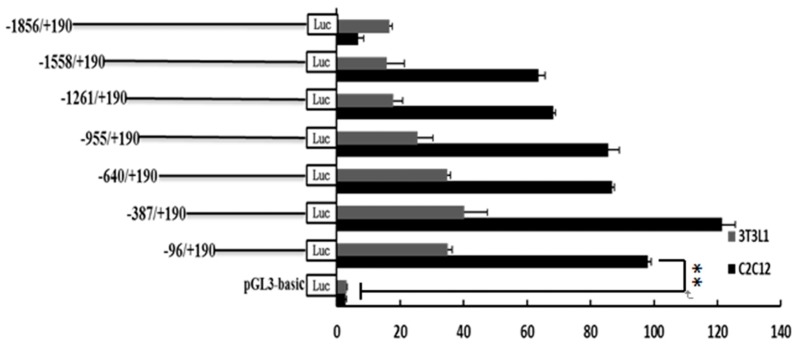
Luciferase activities of the bovine *SLC27A1* promoter constructs in two cell lines. Plasmids with 5′ unidirectional deletions of *SLC27A1* promoter portions were generated (pGL3–1856, 1558, 1261, 955, 640, 387, pGL3–96, and pGL3) and were cloned into a luciferase reporter construct before transfection into C2C12 and 3T3L1 cells, with 48 h allowed to elapse prior to assessing the levels of luciferase activity. Data are means ± SD, with Renilla luciferase activity used for normalization of values. The unpaired Student’s *t*-test was used to detect significant differences. ** *P* < 0.01. Results were replicated in two independent experiments.

In order to assess the potential role that regulatory transcription factors may be playing in governing the *SLC27A1* expression and to identify the minimum sequence needed for gene transcription, we generated seven progressive deletion constructs in which increasingly large regions of the 5′ end of the promoter was absent. These mutant promoter sequences were used in a luciferase reporter assay system following transfection into C2C12 and 3T3L1 cells (Figure 4). The pGL–1558/+190 mutant was associated with up to a 22-fold increase in promoter activation relative to a control promoter-free reporter construct, suggesting that a functional promoter was present in the –1558/+190 region of *SLC27A1*. In the C2C12 cell line, the pGL–1558/+190 construct showed 9.3-fold increased transcriptional activity relative to the pGL-1856/+190 construct, whereas no difference was observed in 3T3L1 cells, suggesting that negative regulators specifically in C2C12 cells were able to bind this region and suppress transcription. No changes were detected in the transcriptional activity when regions from positions −1558 to −96 of the promoter were deleted, and the −96/+190 construct exhibited up to a 34-fold higher transcriptional activity compared to the pGL3-basic construct in C2C12 and 3T3L1 cells (Figure 4). This suggests that the core functional *SLC27A1* promoter is in the −96/+190 region relative to the TSS.

### 3.4. KLF15 Activates the Core SLC27A1 Promoter

**Figure 5:**
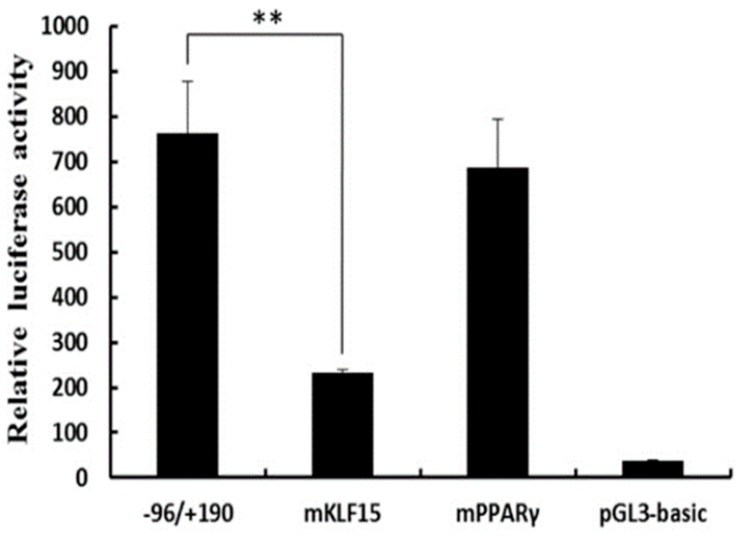
Assessment of KLF15 and PPARγ binding sites via site-directed mutagenesis. The pGL−96/+190 construct were subjected to site-specific mutagenesis and C2C12 cells were transfected with the mutant constructs for 48 h, after which luciferase activity was assessed. Data are means ± SD, with Renilla luciferase activity used for normalization of values. The paired Student’s *t*-test was used to detect significant differences. ** *P* < 0.01. Results were replicated in two independent experiments.

We next generated plasmids in which 3 bp mutations were made within putative transcription factor binding sites in the core *SLC27A1* promoter region. These constructs were then transfected in C2C12 cells. When the KLF15 binding site (−57 to −39) was mutated, there was a ~70% drop in the promoter activity (Figure 5), whereas mutating the PPARγ binding site (−38 to −16) did not alter the promoter activity in these cells. Similarly, promoter activity was unaffected when the binding sites for SREBP (−345 to −331), STAT5B (−296 to −278), KLF15 (−99 to −77), and PPARγ (−108 to −85) in the extended pGL−387/+190 construct were mutated.

### 3.5. KLF15 Binds to the SLC27A1 Promoter

**Figure 6:**
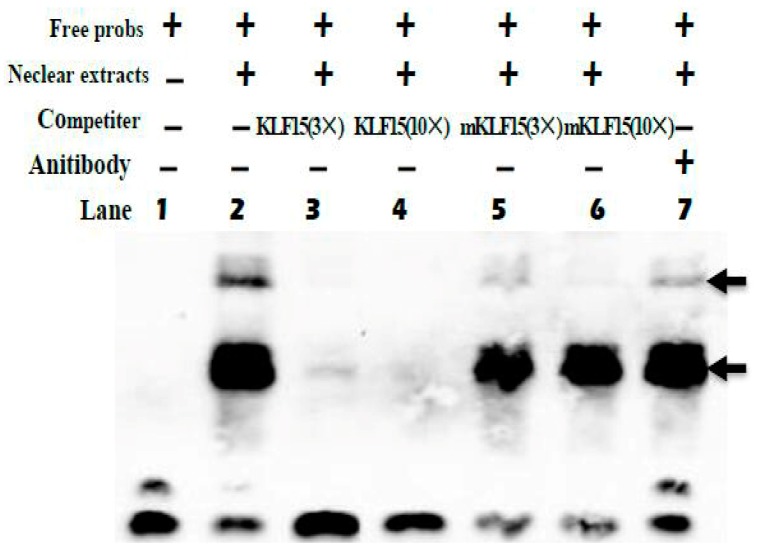
EMSA of KLF15 binding to the *SLC27A1* promoter. Arrows denote primary complexes. KLF15 probes were combined with nuclear extracts as well as 3× unlabeled probe (lane 3), 10× unlabeled probe (lane 4), 3× mutated probe (lane 5), 10× mutated probe (lane 6), or no competition (lane 2). Super-shifting was assessed by addition of 10 ng anti-KLF15 (lane 7).

We next employed an EMSA approach to gauge the ability of KLF15 to directly bind to the promoter of *SLC27A1*. Using biotinylated KLF15 probes, we found that nuclear extracts of C2C12 cells were able to interact with these probes to yield three complexes (lane 2, Figure 6). The specificity of this binding was confirmed using competition assays (lanes 3 and 4, Figure 6), while mutated probes failed to impact these complexes (lanes 5 and 6, Figure 6). When anti-KLF15 was added, a super-shift occurred (lane 7, Figure 6). Chromatin immunoprecipitation using murine muscle tissue similarly confirmed that endogenous KLF15 is enriched at the *SLC27A1* promoter in these animals [17].

## 4. Discussion

Intramuscular fat deposition and the fatty acid profiles of beef are determined mainly by lipid metabolism, which dictates the balance between fat deposition and fat removal in skeletal muscles. In the present study, we observed an elevated expression of *SLC27A1* in tissues with high rates of lipid metabolism including the heart, adipose tissue, and the longissimus thoracis muscle, in line with previous results [8,22,23]. *SLC27A1* is involved in the genetic network for oleic acid synthesis [2], suggesting that *SLC27A1* might be a useful target for regulating the lipid composition of beef [17].

Characterization of the promoter sequence involved the identification of transcription initiation sites. Our data indicate that transcription initiates at a number of sites at the 5′-end of the gene, making exon 1 variable in length. This observation is not consistent with the fact that the gene appears to lack TATA and CCAAT boxes, a condition often associated with multiple TSSs [24]. Vertebrates have been shown to exhibit either focused or dispersed promoters, with the latter arising when several relatively weak TSSs are present within a 100 bp region [25]. For several human genes, dispersed promoters are more common than focused ones [26]. By deleting sections of the *SLC27A1* promoter, we were able to determine that the −96/+190 region surrounding the TSS contained the core promoter, similar to findings in mice and 3T3L1 cells [27].The murine promoter also contains a site where both PPARα and PPARγ can bind in order to control *SLC27A1* expression [28,29]. Oleate uptake increased when 3T3L1 cells were treated with PPARγ activating compounds [28]. In the bovine promoter, however, mutation of the two PPARγ binding sites failed to impact *SLC27A1* promoter activity. KLF15 reportedly induces PPARγ activity to drive adipogenesis [30]. Through EMSA experiments and mutagenesis assays we were able to confirm that KLF15 binds and regulates the *SLC27A1* promoter. This suggests that PPARγ may not directly impact the transcription of this gene, although it may do so indirectly via KLF15. Proximal human *SLC27A1* promoters have been shown to be induced by KLF15 [20], while the human *SLC27A1* promoter was found to be most effectively regulated by KLF15 based on mutational analysis of factors needed for transactivation [14]. Additional bioinformatics/ChIP studies have similarly found that KLF15 plays a key role in regulating many genes associated with lipid flux [17]. KLF15 is also vital to the control of skeletal muscle [16,17], myocardial lipid flux [20], and adipogenic gene regulation [30,31,32,33,34]. The present findings suggest that KLF15 play a key role in controlling *SLC27A1* transcription, thereby potentially regulating bovine skeletal muscle lipid levels.

## 5. Conclusions

In the present study, we determined that KLF15 regulates *SLC27A1* expression, whereas PPARγ does not appear to act directly due to KLF15 mediation. Whether other transcription factors are able to bind the *SLC27A1* promoter and regulate it, remains to be determined. Our findings have provided new insights into *SLC27A1* regulation and its role in regulating beef lipid composition.

## Supplementary Materials

The following are available online at https://www.mdpi.com/2076-2615/9/9/654/s1, Table S1: Primers used in the expression and functional analysis of the *SLC27A1* promoter.

## References

[1] Grundy S.M. (1994). Influence of stearic acid on cholesterol metabolism relative to other long-chain fatty acids. Am. J. Clin. Nutr..

[2] Zhang L., Michal J.J., O’Fallon J.V., Pan Z., Gaskins C.T., Reeves J.J., Busboom J.R., Zhou X., Ding B., Dodson M.V. (2012). Quantitative genomics of 30 complex phenotypes in Wagyu x Angus F (1) progeny. Int. J. Biol. Sci..

[3] Stahl A., Evans J.G., Pattel S., Hirsch D., Lodish H.F. (2002). Insulin causes fatty acid transport protein translocation and enhanced fatty acid uptake in adipocytes. Dev. Cell.

[4] Choi H., Kim S.J., Park S.S., Chang C., Kim E. (2011). TR4 activates FATP1 gene expression to promote lipid accumulation in 3T3-L1 adipocytes. FEBS Lett..

[5] Schaffer J.E., Lodish H.F. (1994). Expression cloning and characterization of a novel adipocyte long chain fatty acid transport protein. Cell.

[6] Wu Q., Ortegon A.M., Tsang B., Doege H., Feingold K.R., Stahl A. (2006). FATP1 is an insulin-sensitive fatty acid transporter involved in diet-induced obesity. Mol. Cell. Biol..

[7] Kim J.K., Gimeno R.E., Higashimori T., Kim H.J., Choi H., Punreddy S., Mozell R.L., Tan G., Stricker-Krongrad A., Hirsch D.J. (2004). Inactivation of fatty acid transport protein 1 prevents fat-induced insulin resistance in skeletal muscle. J. Clin. Investig..

[8] Ordovas L., Roy R., Zaragoza P., Rodellar C. (2006). Structural and functional characterization of the bovine solute carrier family 27 member 1 (SLC27A1) gene. Cytogenet. Genome Res..

[9] Man M.Z., Hui T.Y., Schaffer J.E., Lodish H.F., Bernlohr D.A. (1996). Regulation of the murine adipocyte fatty acid transporter gene by insulin. Mol. Endocrinol..

[10] Abumrad N.A., Forest C.C., Regen D.M., Sanders S. (1991). Increase in membrane uptake of long-chain fatty acids early during preadipocyte differentiation. Proc. Natl. Acad. Sci. USA.

[11] Gimeno R.E. (2007). Fatty acid transport proteins. Curr. Opin. Lipidol..

[12] Ordovas L., Roy R., Zaragoza P., Hayes H., Eggen A., Rodellar C. (2005). Assignment of the solute carrier family 27 member 1 (SLC27A1) gene to bovine chromosome 7. Anim. Genet..

[13] Casas E., Shackelford S.D., Keele J.W., Koohmaraie M., Smith T.P., Stone R.T. (2003). Detection of quantitative trait loci for growth and carcass composition in cattle. J. Anim. Sci..

[14] Jeong J., Kwon E.G., Im S.K., Seo K.S., Baik M. (2012). Expression of fat deposition and fat removal genes is associated with intramuscular fat content in longissimus dorsi muscle of Korean cattle steers. J. Anim. Sci..

[15] Gray S., Wang B., Orihuela Y., Hong E.G., Fisch S., Haldar S., Cline G.W., Kim J.K., Peroni O.D., Kahn B.B. (2017). Regulation of gluconeogenesis by Kruppel-like factor 15. Cell Metab..

[16] Shimizu N., Yoshikawa N., Ito N., Maruyama T., Suzuki Y., Takeda S., Nakae J., Tagata Y., Nishitani S., Takehana K. (2011). Crosstalk between glucocorticoid receptor and nutritional sensor mTOR in skeletal muscle. Cell Metab..

[17] Haldar S.M., Jeyaraj D., Anand P., Zhu H., Lu Y., Prosdocimo D.A., Eapen B., Kawanami D., Okutsu M., Brotto L. (2012). Kruppel-like factor 15 regulates skeletal muscle lipid flux and exercise adaptation. Proc. Natl. Acad. Sci. USA.

[18] Gray S., Feinberg M.W., Hull S., Kuo C.T., Watanabe M., Sen S., DePina A., Haspel R., Jain M.K. (2002). The Krüppel-like factor KLF15 regulates the insulin-sensitive glucose transporter GLUT4. J. Biol. Chem..

[19] Zhao Z.D., Zan L.S., Li A.N., Cheng G., Li S.J., Zhang Y.R., Wang X.Y., Zhang Y.Y. (2016). Characterization of the promoter region of the bovine long-chain acyl-CoA synthetase 1 gene: Roles of E2F1, Sp1, KLF15, and E2F4. Sci. Rep..

[20] Prosdocimo D.A., Anand P., Liao X., Zhu H., Shelkay S., Artero-Calderon P., Zhang L., Kirsh J., Moore D., Rosca M.G. (2014). Kruppel-like factor 15 is a critical regulator of cardiac lipid metabolism. J. Biol. Chem..

[21] Livak K.J., Schmittgen T.D. (2011). Analysis of relative gene expression data using real-time quantitative PCR and the 2(-Delta Delta C(T)) Method. Methods.

[22] Zhao Z.D., Li A.N., Wei S.J., Wang M.M., Li S.J., Zan L.S. (2015). Genetic polymorphisms of the FATP1 gene and their associations with meat quality traits in Chinese. Genet. Mol. Res..

[23] Binnert C., Koistinen H.A., Martin G., Andreelli F., Ebeling P., Koivisto V.A., Laville M., Auwerx J., Vidal H. (2000). Fatty acid transport protein-1 mRNA expression in skeletal muscle and in adipose tissue in humans. Am. J. Physiol. Endocrinol. Metab..

[24] Juven-Gershon T., Hsu J.Y., Kadonaga J.T. (2006). Perspectives on the RNA polymerase II core promoter. Biochem. Soc. Trans..

[25] Juven-Gershon T., Hsu J.Y., Theisen J.W., Kadonaga J.T. (2008). The RNA polymerase II core promoter—The gateway to transcription. Curr. Opin. Cell Biol..

[26] Butler J.E., Kadonaga J.T. (2002). The RNA polymerase II core promoter: A key component in the regulation of gene expression. Genes Dev..

[27] Hui T.Y., Frohnert B.I., Smith A.J., Schaffer J.E., Bernlohr D.A. (1998). Characterization of the murine fatty acid transport protein gene and its insulin response sequence. J. Biol. Chem..

[28] Frohnert B.I., Hui T.Y., Bernlohr D.A. (1999). Identification of a functional peroxisome proliferator-responsive element in the murine fatty acid transport protein gene. J. Biol. Chem..

[29] Martin G., Schoonjans K., Lefebvre A.M., Staels B., Auwerx J. (1997). Coordinate regulation of the expression of the fatty acid transport protein and acyl-CoA synthetase genes by PPARalpha and PPARgamma activators. J. Biol. Chem..

[30] Mori T., Sakaue H., Iguchi H., Gomi H., Okada Y., Takashima Y., Nakamura K., Nakamura T., Yamauchi T., Kubota N. (2005). Role of Kruppel-like factor 15 (KLF15) in transcriptional regulation of adipogenesis. J. Biol. Chem..

[31] Bell-Anderson K.S., Funnell A.P., Williams H., Mat Jusoh H., Scully T., Lim W.F., Burdach J.G., Mak K.S., Knights A.J., Hoy A.J. (2013). Loss of Krüppel-like factor 3(KLF3/BKLF) leads to upregulation of the insulin-sensitizing factor adipolin (FAM132A/CTRP12/C1qdc2). Diabetes.

[32] Small K.S., Hedman A.K., Grundberg E., Nica A.C., Thorleifsson G., Kong A., Thorsteindottir U., Shin S.Y., Richards H.B., Soranzo N. (2011). Identification of an imprinted master trans regulator at the KLF14 locus related to multiple metabolic phenotypes. Nat. Genet..

[33] Birsoy K., Chen Z., Friedman J. (2008). Transcriptional regulation of adipogenesis by KLF4. Cell Metab..

[34] Li D., Yea S., Li S., Chen Z., Narla G., Banck M., Laborda J., Tan S., Friedman J.M., Friedman S.L. (2005). Krüppel-like factor-6 promotes preadipocyte differentiationthrough histone deacetylase 3-dependent repression of DLK1. J. Biol. Chem..

