# Supplementary material for: Transcriptional Regulation of the Bovine Fatty Acid Transport Protein 1 Gene by Krüppel-Like Factors 15
Source: Animals (Basel). 2019 Sep 5;9(9):654. doi: 10.3390/ani9090654 (PMC6769441; doi:10.3390/ani9090654)
Supplement: Supplementary file 1 [file animals-09-00654-s001.pdf]

Table S1.

Primers used in the expression and functional analysis of the *SLC27A1* promoter

Reaction	primer	Primer sequence(5'-3')	Binding region	Size (bp)
qRT-PCR	FATP1 forward	AAGAGCCTGGTCAAGTTCTG	--	239
	FATP1 reverse	TAGGAGTAGTGCCCAAATGC	--	--
	β-actin forward	CATCGGCAATGAGCGGTTCC	--	147
	β-actin reverse	ACCGTGTTGGCGTAGAGGTC	--	--
Promoter cloning	FATP1 promoter forward	CTACTGTGGTGGGCACTTG	-1856/-1837	2046
	FATP1 promoter reverse	TTGTTCCCTGGCTGACCTGGAG	+168/+190	--
	FATP1 promoter forward 1	**GAGCTC**CTACTGTGGTGGGCACTTG	-1856/-1837	2046
	FATP1 promoter forward 2	**GAGCTC**CAATGTGCAGAGGTGAGAG	-1558/-1540	1748
	FATP1 promoter forward 3	**GAGCTC**GCGCTAGTGGTAAAGAACCTG	-1261/-1241	1451
	FATP1 promoter forward 4	**GAGCTC**TCCACTGGAGGAGTACTG	-955/-938	1145
	FATP1 promoter forward 5	**GAGCTC**AAGGCCACAGGATGGGAGGAGAAAG	-640/-616	830
	FATP1 promoter forward 6	**GAGCTC**GCGACAGTGTCAAGCCTCAG	-387/-368	577
	FATP1 promoter forward 7	**GAGCTC**AGAGCTGAGAAGGTCGGCCAAG	-96/-75	286
	FATP1 promoter reverse 1	**CTCGAG**TTGTTCCCTGGCTGACCTGGAG	+168/+190	--
5'RACE	FATP1-GSP1	GAACTGGTCCGAGAACGGGTTGAGG	--	948
	FATP1-GSP2	CGCATAGTCGTGCCTTTCCGGCTTC	--	531
Site-directed mutagenesis	mKLF15 forward	CCAAGCAGGAAAGAAACAAGCACCCGTGGGATAGGCAGGGGGG	-79/-36	286
	mKLF15 reverse	CCCCCCTGCCTATCCCACGGGTGCTTGTTTCTTTCCTGCTTGG	--	--
	mPPARγ forward	GATAGGCAGGGGGGCATTAGTAGGGGGAGCTTGGAG	-50/-15	286
	mPPARγ reverse	CTCCAAGCTCCCCCTACTAATGCCCCCCTGCCTATC	--	--
EMSA	KLF15 forward	CCAAGCAGGAAAGAAACAAGCAGGGGTGGGATAGGCAGGGGGG	-79/-36	286
	KLF15 reverse	CCCCCCTGCCTATCCCACCCCTGCTTGTTTCTTTCCTGCTTGG	--	--
